# The Linkages Between Reimbursement and Prevention: A Mixed-Methods Approach

**DOI:** 10.3389/fpubh.2021.750122

**Published:** 2021-10-27

**Authors:** Ellen Zwaagstra Salvado, Hilco J. van Elten, Erik M. van Raaij

**Affiliations:** ^1^Rotterdam School of Management, Erasmus University Rotterdam, Rotterdam, Netherlands; ^2^Erasmus School of Health Policy and Management, Erasmus University Rotterdam, Rotterdam, Netherlands

**Keywords:** prevention, reimbursement, incentives, primary prevention, secondary prevention, tertiary prevention, quaternary prevention, rapid review methods

## Abstract

**Background:** The benefits of prevention are widely recognized; ranging from avoiding disease onset to substantially reducing disease burden, which is especially relevant considering the increasing prevalence of chronic diseases. However, its delivery has encountered numerous obstacles in healthcare. While healthcare professionals play an important role in stimulating prevention, their behaviors can be influenced by incentives related to reimbursement schemes.

**Purpose:** The purpose of this research is to obtain a detailed description and explanation of how reimbursement schemes specifically impact primary, secondary, tertiary, and quaternary prevention.

**Methods:** Our study takes a mixed-methods approach. Based on a rapid review of the literature, we include and assess 27 studies. Moreover, we conducted semi-structured interviews with eight Dutch healthcare professionals and two representatives of insurance companies, to obtain a deeper understanding of healthcare professionals' behaviors in response to incentives.

**Results:** Nor fee-for-service (FFS) nor salary can be unambiguously linked to higher or lower provision of preventive services. However, results suggest that FFS's widely reported incentive to increase production might work in favor of preventive services such as immunizations but provide less incentives for chronic disease management. Salary's incentive toward prevention will be (partially) determined by provider-organization's characteristics and reimbursement. Pay-for-performance (P4P) is not always necessarily translated into better health outcomes, effective prevention, or adequate chronic disease management. P4P is considered disruptive by professionals and our results expose how it can lead professionals to resort to (over)medicalization in order to achieve targets. Relatively new forms of reimbursement such as population-based payment may incentivize professionals to adapt the delivery of care to facilitate the delivery of some forms of prevention.

**Conclusion:** There is not one reimbursement scheme that will stimulate all levels of prevention. Certain types of reimbursement work well for certain types of preventive care services. A volume incentive could be beneficial for prevention activities that are easy to specify. Population-based capitation can help promote preventive activities that require efforts that are not incentivized under other reimbursements, for instance activities that are not easily specified, such as providing education on lifestyle factors related to a patient's (chronic) disease.

## Introduction

Healthcare prevention, ranging from regular dental cleaning to collective initiatives to promote a healthier lifestyle, is one of the most important pillars of public health (1). Major gains in health can be accomplished through prevention (2). Moreover, prevention has the potential to substantially reduce disease's economic burden (3), especially in the current environment of growing chronic illness (4). Healthcare prevention focuses on promoting and protecting people's health by ensuring they receive care that conforms to their needs and stage of disease (5). While primary, secondary, and tertiary prevention focus on delivering care to avoid disease onset, allow early diagnose and reduce disease impact, respectively (6), quaternary prevention aims to protect patients from receiving redundant, unnecessary care (7). Healthcare professionals face the challenge of having to promptly assess a patient's need for preventive interventions (8, 9).

This crucial deliberation could, however, be disrupted by incentives in reimbursement systems (10). In healthcare systems with a purchaser-provider split, third-party funders such as insurers or the government can pay professionals for the services provided based on different types of reimbursement schemes (11). Reimbursement schemes can vary on many aspects, such as unit of payment (e.g., per service, per patient, or per day), payment amount, or timing (11). Different combinations of these characteristics are argued to create different (financial) incentives that promote or hinder professionals' behavior in everyday practice, e.g., providing less or more services than necessary (10, 11). As for pay-for-performance (P4P), one of its reported perverse incentives is that it might focus providers' attention to what is being measured, and consequently marginalize other quality criteria that are not being rewarded (12).

All-in-all, reimbursements have a well-documented reputation for incentivizing unwanted behavior. However, in the same train of thought, well-designed reimbursement schemes may allow the possibility to incentivize the behavior we do want, i.e., to focus professionals on prevention. Therefore, reimbursement schemes may play an important role in supporting meaningful prevention (13).

Much research has been devoted to investigating how various reimbursement schemes (and their respective incentives) impact healthcare delivery (11, 14–16). However, the existing body of literature still lacks comprehensive research on the impact of reimbursement schemes on professionals' behavior on all four levels of prevention. This study addresses this gap by incorporating evidence from both a rapid review and original empirical research, to address our research question: How do different types of reimbursement schemes in healthcare affect healthcare professionals' behavior in terms of the delivery of prevention?

## Methods

To address our research question, we use a mixed-methods approach (17). We conduct both a rapid literature review for a broad overview of the literature and semi-structured interviews with healthcare professionals for more in-depth insights. In this section, we present the research methods.

### Rapid Review

We review the literature using a rapid review methodology. With more widely established systematic reviews, time and resource consumption may pose as barriers for its use in strategic decision making and health policy formulation. Rapid reviews are known for providing information on a specific research topic within a limited timeframe, applying systematic review methodology with explicitly stated shortcuts whilst maintaining rigorous methodology (18). The shortcuts applied to tailor our rapid review are specifically stated as the use of one database and one main reviewer. As progress toward universal health coverage should be informed by timely evidence, rapid reviews are an efficient approach to producing relevant evidence often to support decision-makers and strengthen health policies (19).

For our review, studies were systematically identified using the online database PubMed. Only English language scientific articles were considered for which full text was available, published from 2010 up until April 25th 2020, using the Pubmed “Humans” filter. Different search strings were tested using five previously identified relevant papers. To ensure that the relevant studies were included, the final search string was achieved based on the results of this testing. The search string can be found in the Supplementary Material.

The inclusion and exclusion criteria were defined *a priori*. All types of academic primary research studies (empirical studies and conceptual works) were considered. Editorials, systematic reviews, and studies reporting and/or commenting on data from other studies were excluded. The population of interest is healthcare professionals, broadly defined as qualified medical professionals who deliver primary, secondary, and/or tertiary healthcare services. Therefore, the search string contains various terms that are used to describe different types of healthcare professionals. As previously described, it is expected that reimbursement schemes and payment models induce a variety of behaviors in professionals, thus impacting the delivery of prevention. The phenomenon of interest comprises reimbursement schemes' effect on primary, secondary, tertiary and/or quaternary prevention. Professionals' behaviors expressed in process and/or outcome measures were included as long as it pertained to primary, secondary, tertiary and/or quaternary prevention. Studies that do not define the specific type of reimbursement under analysis were excluded, as well as studies that analyze the effect of reimbursement on prevention in combination with other interventions without isolating the effect of reimbursement. For example, a study from Kalwij et al. (20) examines the impact of financial incentives combined with practice-based support (audits and feedback) on performance and on screening behavior, instead of the isolated effect of financial incentives. This led to its exclusion during full text screening.

**Figure 1:**
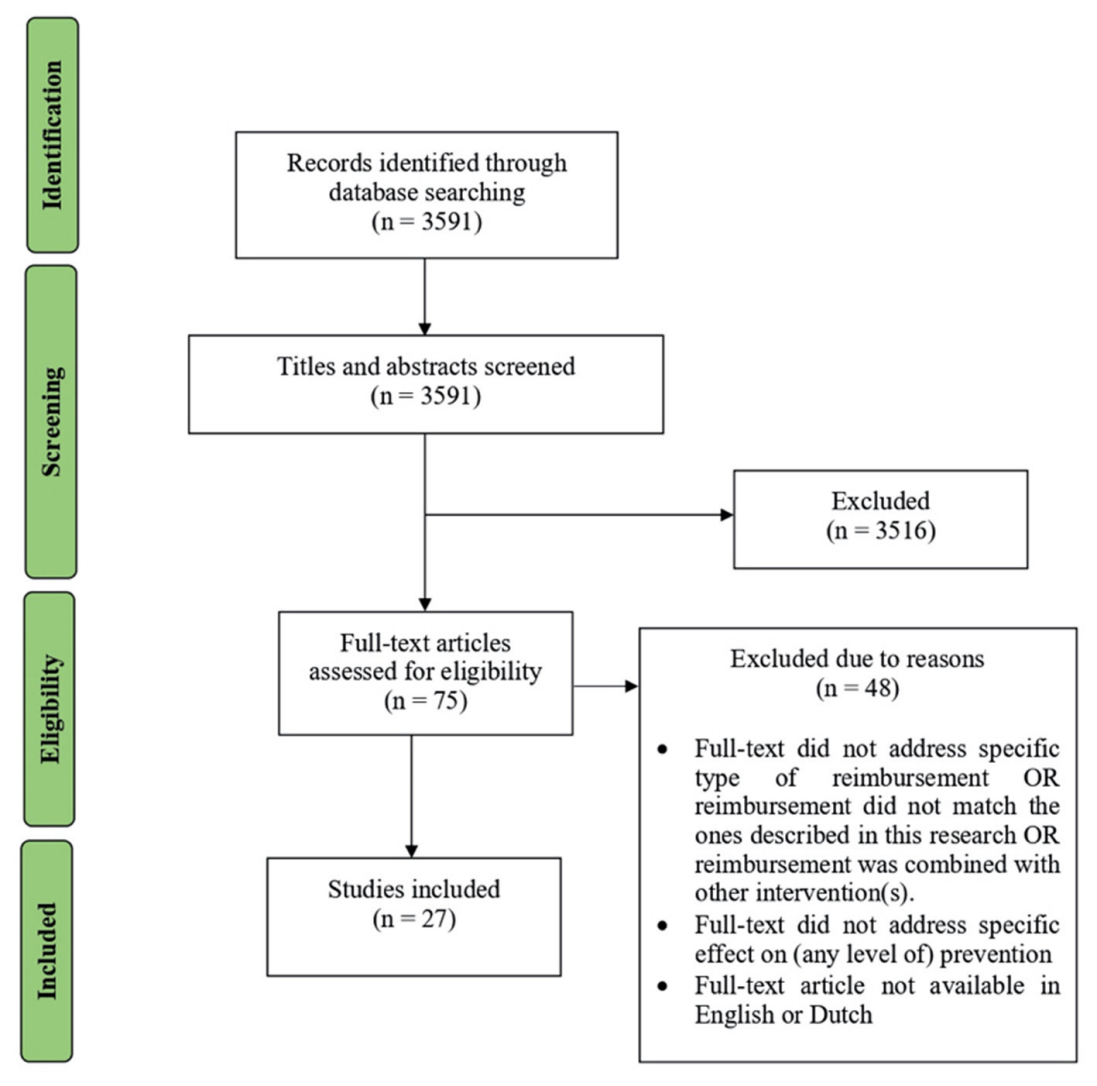
Flow diagram of inclusion and exclusion process based on PRISMA flow diagram (21).

Our rapid review's search string yielded 3,591 papers from PubMed. Figure 1 illustrates the inclusion and exclusion process. One author (ES) screened titles and abstracts. This resulted in 75 papers eligible for full-text screening. Full-text screening led to the exclusion of 48 studies due to reasons such as type of reimbursement is not specified, or link to prevention is not clear. Each step in this process was discussed within the author team, before as well as during execution of each step. A total of 27 conceptual or empirical papers published between January 2010 and April 2020 were included for the qualitative synthesis, all related to the effect of reimbursement of healthcare professionals on delivery of prevention.

Two authors (HE and ER) provided feedback and assisted in calibrating eligibility criteria, screening and selecting papers, and cross-checking data extraction. The relevant data from all included studies were extracted and collected in an Excel spreadsheet. These results were posteriorly synthesized according to the type of reimbursement and prevention level(s) addressed and analyzed in terms of the relationships between reimbursement types and preventive behaviors.

### Semi-structured Interviews

We conducted semi-structured interviews to further our understanding of the subject matter. We chose this qualitative method to collect in-depth data and capture meanings and perceptions people attribute to a certain phenomenon (22). In conjunction with the rapid nature of our research and limited by COVID-19 restrictions, our number of interview participants is limited. In total, ten semi-structured in-depth interviews were conducted with eight Dutch healthcare professionals—consisting of four general practitioners (GP) and four physical therapists (PT)—as well as two members of the prevention and purchasing departments of Dutch insurance companies (representing the payers in the Dutch healthcare system). An overview of respondents' characteristics can be found in the Supplementary Material.

In the Netherlands, GP practices are reimbursed through a 3-segment funding model. In the first segment, GPs are reimbursed through a mix of capitation and FFS for primary care activities provided. The second segment consists of funding through episode-based payments where GPs receive a fixed fee for every patient for which they provide multidisciplinary care such as diabetes care and other selected chronic diseases. In segment 3, GPs and insurance companies have the opportunity to negotiate additional P4P contracts and GPs become eligible to receive a bonus for reaching certain outcomes (at practice level) pertaining to the care delivered in segments 1 and 2.

Physical therapy in the Netherlands is reimbursed under FFS, in other words, paid according to the number of physical therapy sessions. Practices are free to make specific agreements with purchasers regarding delivery of care, volume or outcomes in exchange for financial rewards. Professionals under different types of reimbursement were purposely selected and answers were analyzed according to the type of reimbursement.

All semi-structured interviews were held between March and April 2020 and were conducted via telephone due to the COVID-19 outbreak. Interviews were recorded and transcribed verbatim in Dutch and the quotes used in this manuscript were posteriorly translated to English. Interviews were coded and analyzed using software ATLAS.ti, version 8.4.4. The interview transcripts were analyzed first using open coding and subsequently using axial coding to integrate codes into categories and identify relationships between categories. Examples of codes include “Obstacles for prevention,” “Efforts made toward prevention,” “Perceptions about own role in prevention” and “Strategies to mitigate overmedicalization.” The code group list can be found in the Supplementary Material.

As previously mentioned, qualitative research allows for the collection of in-depth data such as perspectives and perceptions; elements that would be much more difficult to obtain from quantitative data (22). However, these essential elements that provide an extra dimension and enrich findings of qualitative research are also the subject of controversies regarding quality and trustworthiness of its results. Therefore, besides the reliance on multiple research methods, we have incorporated other strategies in this research to enhance its validity and reliability. Concerning the empirical part of this research, interviews were recorded to ensure descriptive validity and increase reliability. The use of in-depth open interviews helps mitigate interpretation bias and consequently increase internal validity. Interviews were transcribed *verbatim* and transcripts were made available to increase internal reliability. The use of a topic list to guide interviews helped mitigate researcher bias regarding assumptions or beliefs that might otherwise have compromised validity. The topic list can be found in the Appendix. The interviewing process as well as data collection, analysis and interpretation steps were discussed between the authors and are described in detail allowing for a well-documented audit-trail of materials and processes. Regarding our rapid review, we tested different search strings in order to find the search string that yields as many relevant studies and thus achieve higher sensitivity.

## Results

In this section, the research findings are presented. We present the rapid review's findings organized by type of reimbursement scheme and complement these with relevant findings from the semi-structured interviews.

The rapid review yielded 27 studies; their respective characteristics are presented in more detail in Table 1. Another table with more detailed information about the included studies can be found in the Supplementary Material. The reviewed studies were executed in a variety of countries and delivery models. More than half (14 out of 27) in a country with a National Health Insurance (NHI) model: eight in Canada (27, 29, 35, 36, 38, 40, 41, 46), five in Taiwan (24, 25, 31, 37, 44), and one in Rwanda (28). A further five in a country with a Beveridge model: four from the UK (34, 39, 43, 48) and one from Italy (32). Four studies were executed in the US, with their focus varying from publicly funded safety-net community health centers (30), Medicaid-focused managed care (26), a commercial health plan (23) to a cross-sector study (45). Two studies in a country with a Bismarck model: one in Estonia (42) and one in France (49). One study was executed in Mozambique and concerned a donor-sponsored program (47). Finally, one study (33) analyzed data from 14 different European countries, including countries with a Bismarck model (such as The Netherlands) and countries with a Beveridge model (such as Sweden).

**Table 1:** Overview of included studies in rapid review.

**Reference**	**Study design**	**Country/delivery model**	**Level of prevention**	**Reimbursement**	**Aims of study**	**Care domain**	**Primary findings**
Chen et al. (23)	Longitudinal retrospective study	USA Commercial Health Plan (not government-run)	Secondary Tertiary	P4P Bonus (3.5% above reimbursement: maximum $16,000/year). Performance based on physician level	To assess the impact of the P4P program on improved quality of care (lipid monitoring and treatment) and quality of care on outcomes (new coronary events, hospitalizations, and lipid control) for cardiovascular disease.	Primary care setting and medical specialized care	P4P was associated with a higher likelihood of receiving quality care (OR = 0.70; 95% CI = 0.54–0.90) compared to non-P4P. Receiving quality care was then associated with a lower likelihood of new coronary events (OR = 0.80; 95% CI = 0.69–0.92), hospitalization (OR = 0.76; 95% CI = 0.69–0.83), or uncontrolled lipids (OR = 0.67; 95% CI = 0.61–0.73), *p* < 0.01.
Chen et al. (24)	Controlled before and after study	Taiwan NHI model	Secondary Tertiary	P4P Bonus for completion of visits $3/visit/patient + additional bonus for further screening, referral, early detection of abnormalities per patient ($15–$30). Performance based on physician level	To evaluate the effect of a P4P program targeting providers' performance on three indicators for Hepatitis B and C guideline-recommended preventive services.	Hospital and clinic physicians	P4P was associated with a significantly higher likelihood of receiving all three recommended services (OR = 1.13; 95% CI: 1.07–1.19). The before and after difference between the two groups was modest (5–23%).
Cheng et al. (25)	Population-based natural experiment	Taiwan NHI model	Secondary Tertiary	Episode-based vs. FFS	To examine the impacts of diagnosis-related group (DRG) payments on health care provider's behavior (medical service content and healthcare outcomes) compared to the traditional FFS.	Hospital setting	DRG payment resulted in a decrease of 10% (p < 0.001) in length of stay in the intervention group in relation to the comparison group. The number of orders that define intensity of care declined significantly (*p* < 0.001) with differences of 1.230, 2.695, and 1.070 items. No significant changes were found at *p* < 0.001 significance level for healthcare outcomes variables.
Chien et al. (26)	Case-comparison and interrupted times series	USA Not-for-profit Medicaid-focused managed care plan	Primary	P4P Piece-rate bonus (15–25% above reimbursement) Bonus for immunizations $100/patient and extra bonus $100/patient for timeliness. Performance based on practice level	To evaluate the impact of a “piece-rate” P4P program on fully and up-to-date immunization of 2-year-olds.	All types of healthcare practices (not further specified)	Results on the fourth year of intervention show that the P4P healthcare program presented significantly (OR = 0.60, SE = 0.12, *p* < 0.01) higher fully and timely immunization rates than the comparison group.
Dahrouge et al. (27)	Cross-Sectional study	Canada NHI model	Primary Secondary	FFS vs. Salary vs. New capitation (Capitation + 10% FFS) vs. Traditional capitation	To compare delivery of preventive services (immunizations and screenings) by practices under four different primary care funding models and to identify organizational factors associated with superior preventive care.	Primary care (family health networks; health services organizations; community health centers)	After adding physician characteristics and organizational structure factors in multilevel regression analysis, reimbursement was (no longer) statistically significant. Having at least one female family physician (β = 8.0, 95% CI 4.2–11.8), a panel size of fewer than 1,600 patients per FTE family physician (β = 6.8, 95% CI 3.1–10.6) and an electronic reminder system (β = 4.6, 95% CI 0.4–8.7) were more significant.
De Walque et al. (28)	Prospective quasi-experimental study	Rwanda NHI model	Primary secondary	P4P Bonus (different amounts for different services: individual testing US$0.92; couple testing US$4.59). Performance based on practice level	To examine the impact of a P4P incentive on two output indicators: individual and couples HIV testing and counseling.	Healthcare Facilities (not further specified)	The impact of P4P on HIV testing and counseling was significant for individuals living in couple (β (estimated effect) = 0.102, SE = 0.041, *p* = 0.012) and for discordant couples (β = 0.147, SE = 0.068 *p* = 0.0130) and not significant for individuals not living in couple (β = 0.003, SE = 0.062, *p* = 0.959) and not discordant couples (β = 0.072, SE = 0.070, *p* = 0.304).
Échevin and Fortin (29)	Natural experiment	Canada NHI model	Secondary Tertiary	Per-diem	To examine the impact of hospital specialists' reimbursement on length of stay (LOS) and re-hospitalization post-discharge as an alternative to traditional FFS.	Specialist physicians at hospital setting	Under the new per-diem reimbursement there was an increase in the LOS by 0.28 days (SE = 0.07) corresponding to 4.2% increase. The reform did not impact the risk of re-hospitalization at a global level (β = −12,798, *p* > 0.202).
Gavagan et al. (30)	Retrospective analysis of a natural quasi-experiment	USA Community Health Program (CHP) Clinics	Primary Secondary	P4P Bonus [max $12,000 annually/physician ($4,000/target = 3–4% annual income)]. Performance based on physician level	To evaluate physician P4P program on quality of preventive care (childhood immunization and cervical- and breast cancer screenings).	Primary care (community health centers)	P4P was associated with slight improvements in performance for mammography (*p* = 0.076) and cervical cancer screenings (*p* = 0.053, however this was not considered clinically significant. The effect on immunizations was not significant (*p* = 0.79). Survey results point out that physicians felt the incentives were not very effective in improving quality of care.
Hsieh et al. (31)	Longitudinal cohort study	Taiwan NHI model	Secondary Tertiary	P4PDiabetes program. Phase 1–process indicators–Bonus for process indicators ($30.00–$75.00/visit); Phase 2–Bonus ($30.00/visit) for process indicators conditional on performance of outcome indicators. Ranking (top 25% performing physician get extra bonus). Performance based on physician level	To examine if a change in P4P (from a program with process measures to process and outcome measures) had impact on diabetes outcomes.	Medical specialized care (hospitals and clinics)	The provision of tests for HbA1c [0.001, 95% CI = (0.000–0.003) *p* = 0.154] and LDL [0.019, 95% CI = (−0.017–0.055) *p* = 0.302] did not significantly differ between both phases. Blood pressure examinations significantly increased [0.068, 95% CI = (0.032–0.103) *p* < 0.001] between phases. Adding outcome measures in the second phase led to significant improvement in HbA1c [−3.135 95% CI = (−3.818–2.453) *p* < 0.001] and LDL levels [−4.323 95% CI = (−6.004–2.643) *p* < 0.001].
Iezzi et al. (32)	Longitudinal cohort study	Italy Beveridge Model	Secondary Tertiary	P4P “low powered incentives”. Performance based on physician level	To analyze the impact of a low powered P4P incentive on diabetes management. The outcome measure is set on the number avoidable hospitalizations.	Primary care (general practitioners)	The results (available upon request) associated financial incentives from P4P with a lower likelihood of experiencing avoidable hospitalizations for diabetes-related diseases.
Jusot et al. (33)	Cross-sectional study	14 European countries Various health delivery models	Primary Secondary	FFS vs. capitation vs. salary	To examine the variations in utilization of preventive services (immunization and screenings) in 14 European countries. One of the health system supply determinants being remuneration methods for physicians.	Entire healthcare system	FFS was associated with a higher probability for colon cancer screening (OR = 3.038 significant at 1%) compared to capitation (OR = 0.593) or salary (OR = 0.395 significant at 1%). Similar results were presented for flu vaccinations. The results associate capitation with the lowest provision of eye exams (OR = 0.493 significant at 1%) while FFS was associated with the highest score (OR = 2.084 significant at 1%).
Karunaratne et al. (34)	Prospective longitudinal cohort study	UK Beveridge model	Tertiary	P4P Quality and Outcomes Framework (QOF) (representing 25% of income). Performance based on practice level	To evaluate the effectiveness of adding renal indicators to P4P program on hypertension management in primary care patients with chronic kidney disease (CKD) by analyzing changes in recorded blood pressure and prescription patterns before and after their introduction.	Primary care (general practices)	In general, blood pressure (BP) reduced between period 1 and 2 and was sustained in period 3. There was a more pronounced effect in the hypertensive patients (both CKD and not) as mean BP went from 146/79 mmHg to 140/76 in the first 2 years post-P4P (*p* < 0.01) and was sustained in the last 2 years of the study [139/75 (*p* < 0.01)]. Within the hypertensive group the CKD patients had a BP greater reduction, the % of patients with BP reduced to 145/85 mmHg went from 28 to 45.1 to
							55.6%. The BP reduction was associated with an increase in medication prescription and consequently increased prescription costs from €444.726 to €655.842).
Kiran et al. (35)	Longitudinal study	Canada NHI model	Secondary	P4P Bonus (max $8.400/annually−3% of gross income) for reaching screening targets. Performance based on physician level	To assess whether the introduction of a P4P reimbursement scheme was associated with increased cancer screening rates and also its effect on physician payments.	Primary care—PCMH (patient-centered medical homes)	No significant change was found for screening rates after introduction of P4P. E.g., Colon cancer screening rate changed from 3.0% (95% CI, 2.3–3.7%) to 4.7% (95% CI, 3.7–5.7%). Financial incentives for cervical, breast, and colorectal cancer screening accounted for $28.3, $31.3, and $50.0 million expenses respectively between 2006 and 2010.
Kiran et al. (36)	Longitudinal study	Canada NHI model	Secondary Tertiary	Different combinations of FFS and capitation	To understand the effect of each payment model on chronic disease management and prevention (cancer screenings) over time, comparing the effectiveness of different models.	Primary care—PCMH (patient-centered medical homes)	Compared to enhanced fee-for-service, team-based capi-tation was associated with a higher likelihood of performing dia-betes monitoring (39.7 vs. 31.6%, adjusted RR = 1.22, 95% CI = 1.18–1.25), mammography (76.6 vs. 71.5%, adjusted RR = 1.06, 95% CI = 1.06–1.07) and colorectal cancer screening (63.0 vs. 60.9%, adjusted RR = 1.03, 95% CI = 1.02–1.04). Over time, absolute difference in improvement in diabetes monitoring of team-based capitation compared with enhanced fee for ser-vice [10.6% [95% CI 7.9–13.2%)] and with non–team-based capitation [6.4% (95% CI 3.8–9.1%)]. Absolute difference in improvement in cervical cancer screening of team-based capitation com-pared with enhanced fee for service [7.0% (95% CI 5.5–8.5%)] and compared with non-team-based capitation [5.3% (95% CI 3.8–6.8%)]. No significant differ-ences over time for breast and colorectal cancer screening rates.
Lai and Hou (37)	Cross-Sectional study	Taiwan NHI model	Secondary Tertiary	P4P Diabetes Program. Physicians receive fees for enrolling patients in program + Incentives for process indicators and Outcome indicators. Performance based on physician level	To examine the effect of a diabetes mellitus P4P program (DM-P4P) on guideline adherence for diabetes mellitus disease management according to physician participation status. Patients were divided in three groups: patients enrolled in the DM-P4P program, patients not enrolled but treated by DM-P4P-participating physicians, and patients treated by non-P4P physicians.	Physicians (hospitals and practices)	DM-P4P program was associated with a higher likelihood of receiving all 7 guideline-recommended tests/examinations (*p* < 0.001). Patients who were not enrolled in the program but who were treated by DM-P4P-participating physicians were significantly more likely to receive 3/7 of the recommended tests/examinations lipid profile [adjusted RR = 1.24 95%CI = (1.04–1.45)] (*p* < 0.05), ALT [adjusted RR = 1.06 95% CI = (1.00–1.11)] (*p* < 0.1) and eye examination [adjusted RR = 1.21 95%CI = (1.11, 1.31)] (*p* < 0.01) than those treated by non-P4P physicians.
LeBlanc et al. (38)	Longitudinal study	Canada NHI model	Secondary Tertiary	P4P Bonus (annually CAN$83.83/patient for completing all indicators). Performance based on physician level	To study the influence of a P4P program for GPs on the glycemic control of diabetes patients (diabetes disease management).	Primary care (not further specified)	Diabetes patients for which a GP claimed the incentive had greater odds of receiving at least 2 glycemic tests per year (OR = 1.92, 99% CI = 1.87–1.96, *p* < 0.0001) compared to incentive not claimed. These odds increased by 56% (OR = 1.56 99% CI = 1.49–1.62, *p* < 0.0001) following the P4P implementation. No difference in glycemia values between incentive claimed (7.4% SD = 1.4) and incentive not claimed (7.5% SD = 1.4) groups.
Lee et al. (39)	Retrospective cohort study: interrupted time series	UK Beveridge Model	Secondary Tertiary	P4PQOF (representing 25% of income). Performance based on practice level	To evaluate if the P4P program resulted in a step change the quality of care (blood pressure and cholesterol controls) for coronary heart disease, stroke and hypertension.	Primary care (general practices)	The P4P program was associated with an initial trend change pertaining to reduction in systolic blood pressure for hypertension patients (−0.83, CI = −1.08, −0.58) (significance at 1%) compared to the period before implementation. These improvements appear to stabilize in the following years.
Li et al. (40)	Natural experiment	Canada NHI model	Primary Secondary	P4P Bonus for reaching all 5 targets = $11.000 + bonus for scheduling appointments for eligle patients = $11.000 - TOTAL (maximum ($22.000) <10% annual revenue). Performance based on physician level	To identify the impact of a P4P incentive on the provision of five preventive primary care services (immunizations and screenings).	Primary care (primary care physicians)	P4P had a statistically significant effect on the provision of adult immunizations (0.028 SE = 0.007), Pap smears (0.041 SE = 0.005), mammograms (0.018 SE = 0.005), and colorectal cancer (0.085 SE = 0.007) (significant at 1%) leaving only the effect on toddler immunizations non-statistically significant. Representing an increase of 5.1, 7.0, 2.8, and 57% respectively over the base compliance levels.
Liddy et al. (41)	Cross-Sectional study	Canada NHI model	Secondary Tertiary	FFS (mainly FFS) vs. Blended Capitation (mainly capitation) vs. Salary	To compare different primary care models regarding the adherence to ten evidence-based guidelines pertaining to cardiovascular disease management.	Primary care (not further specified)	Diabetes care: significantly higher for salaried professionals than fee-for-service practices [Adjusted OR = 2.4 (95% CI 1.4–4.2), *p* = 0.001]. Smoking cessation drug prescription: Blended capitation practices significantly more likely than salaried professionals [AOR = 2.4 (1.3–4.6), *p* = 0.007]. Weight management measurements: Blended capitation practices were significantly more likely to measure waist circumference than FFS practices [19 vs. 5%, AOR = 3.7 (1.8–7.8), *p* = 0.0006]. No significant difference between models for chronic kidney disease, dyslipidemia, and hypertension management.
Merilind et al. (42)	Interrupted time series	Estonia Bismarck model	Primary	P4P (2–4% of GPs reimbursement). Performance based on physician level	To compare childhood immunization rates of Estonian family doctors joined and not joined the P4P program.	Primary care (family physicians)	There was an improvement in both groups during the observation period, however doctors joined to the quality system met the 90% vaccination criterion more frequently compared to doctors not joined to the quality system. Doctors not joined to the quality system were below the 90% vaccination criterion in all vaccinations listed in the Estonian State Immunization Schedule.
Norman et al. (43)	Qualitative semi-structured interviews	UK Beveridge model	Quaternary	P4P QOF Bonus for quality targets pertaining clinical care, practice organization and patient experience (representing	To examine how GPs experience the British P4P program regarding its consequences for their professional ethos.	Primary care (general practices)	Professionals' opinions on P4P's effect on quaternary prevention: P4P (QOF) has the potential to medicalize pre-disease states and risk factors raising concern about
				25% of income). Performance based on practice level			over-medicalization. The general trend is to introduce medication early on. Incentives have the power to change doctors' behavior and adapt their practices.
Pan et al. (44)	Retrospective cohort study	Taiwan NHI model	Secondary Tertiary	P4P Diabetes Program Performance is based on 4 indicators–final achievement grade places physician in ranking. Top 25% receive additional bonus. Performance based on physician level	To explore the differences in physician continuity of care and survival rates between P4P participants and non-participants diabetes patients.	Medical specialized care (hospitals and clinics)	P4P participation was associated with a higher continuity of care score (COCI) (β = 0.227 (SE = 0.001) (*p* < 0.001) compared to nonparticipants. P4P participants had a lower hazard ratio HR of mortality 0.43 (95% CI = 0.41–0.44, *p* < 0.001).
Pearson et al. (45)	Cross-Sectional study	USA non-federally employed physicians in private offices AND community health centers throughout the US	Primary Secondary Tertiary	Different levels of capitation	To determine whether four different levels of capitated payment were associated with patient education being included more frequently compared to other payments.	Primary care (not further specified)	The likelihood of visits including patient education for different levels of capitation (95%CI): <25% capitation: OR = 1.00(1.00–1.00); 25–50% capitation OR = 0.77 (0.38–1.58); 50–75% capitation OR = 0.81 (0.53–1.25); >75% capitation OR = 3.38 (1.23–9.30).
Pendrith et al. (46)	Quasi experiment	Canada NHI model	Secondary	P4P (FFS vs. FFS + P4P vs. Capitation + P4P) Bonus [$220 (60%)–$2,200 (80%)]. Performance based on physician level	To compare cervical cancer screening rates among three reimbursement models and to estimate the average and marginal costs of screening/patient.	Primary care (not further specified)	The mean adjusted screening rates per reimbursement (*p* < 0.0001): (FFS + P4P) 7.7% (95%CI = 7.6, 7.7) higher compared to FFS and 2.3% (95% CI = 2.3, 2.3) higher compared to (Capitation + FFS) (Capitation + FFS). 6.2% (95% CI = 6.2, 6.3) higher than FFS. GPs practicing in (FFS + P4P) and (capitation + P4P) had significantly higher screening rates compared to FFS alone.
Rajkotia et al. (47)	Retrospective case control	Mozambique Program sponsored by NGO	Primary Secondary Tertiary	P4P Bonus ($0.10–$11.20 per target per patient). Performance based on practice level	To evaluate the effects of P4P program in two provinces (North and South) on the provision of 18 HIV and maternal/child HIV preventive services compared to input-based financing.	Health facilities (not further specified)	P4P was associated with an increase of 251.6% [β = 9.1 (SE = 1.3, *p* < 0.001)] in HIV-infected pregnant women receiving therapy in the North and an increase of 194.6% [19.4 (SE 3.8, *p* < 0.001] in the South relative to the control group. P4P program was associated with significant improvements of 14 indicators in the North and 9 indicators in the South achieving similar improvements. Indicators were not sensitive to price, but rather to the level of effort associated.
Serumaga et al. (48)	Interrupted time series	UK Beveridge model	Secondary Tertiary	P4P QOF (representing 25% of income). Performance based on practice level	To access the impact of P4P incentive on the delivery of quality of care and outcomes among UK patients with hypertension.	Primary care (general practices)	No changes attributed to P4P pertaining to: Blood pressure monitoring (level change = 0.85, 95% CI = −3.04, 4.74, *p* = 0.669 and trend change = −0.01, 95%CI = −0.24,0.21, *p* = 0.615). Blood pressure control (level change = −1.19, 95%CI = −2.06, 1.09, *p* = 0.109 and trend change = −0.01, 95%CI = −0.06, 0.03, *p* = 0.569). Treatment intensity (level change = 0.67, 95% CI = −1.27, 2.81, *p* = 0.412 and trend change = 0.02, 95%CI = −0.23, 0.19, *p* = 0.706) P4P had no effect on the cumulative incidence of stroke, myocardial infarction, renal failure, heart failure, or all cause mortality in both treatment experienced and newly treated subgroups.
Sicsic and Franc (49)	Quasi-Natural experiment	France Bismarck model	Secondary	P4P Bonus–maximum €245/target (80% screened). Performance based on physician level	To study the impact of a P4P program on breast cancer screening.	Primary care (general practitioners)	The probability of undergoing breast cancer screening 1.38 % (95 % CI = 0.41–2.35), did not significantly differ following the implementation of the P4P program.

*OR, odds ratio; CI, confidence interval; RR, relative risk; SE, standard error*.

As presented in Table 1, a total of 20 studies focus on the relationship between P4P bonuses and prevention (23, 24, 26, 28, 30–32, 34, 35, 37–40, 42–44, 46–49). Seven papers study P4P incentives awarded at practice level (26, 28, 34, 39, 43, 47, 48), from which four studies pertain to the Quality and Outcome Framework (QOF) P4P program in the UK, where incentives represent up to 25% of annual income (34, 39, 43, 48). In one study, an extra bonus is awarded to practices that, besides achieving the targets, also manage to do this within a short period of time (26).

The remaining 13 studies on P4P consider bonuses directed at individual professionals (23, 24, 30–32, 35, 37, 38, 40, 42, 44, 46, 49), from which four studies specify that incentives represent between 2 and 4% of professional's income (23, 30, 35, 42) and 1 < 10% (40). Two studies (31, 44) consider a program where an additional bonus (on top of P4P for achieving targets) is awarded to professionals who rank in the top 25%.

From all 20 studies considering P4P, eight studies do not further specify bonus characteristics besides bonus amount and if directed at practice or at individual level (24, 28, 32, 37, 38, 46, 47, 49).

From the remaining seven studies that do not address P4P (25, 27, 29, 33, 36, 41, 45), four studies compare the impact of multiple payment models (FFS vs. salary vs. capitation) (27, 33, 41), or different blends of FFS, capitation and incentives (36), on professionals' behavior toward prevention. One study evaluates the effect of (different levels of) capitated payments (45), another studies a mix of per-diem reimbursement with FFS as an alternative to pure FFS (29), and one other study compares episode-based payments to FFS (25). In these seven studies, FFS reimbursement is used as the benchmark against which other payment models are compared with respect to one or more outcome measures that capture preventive behaviors.

From the 27 studies, the majority (n=16) pertains exclusively to the preventive behaviors of primary care professionals/practices (27, 30, 32, 34–36, 38–43, 45, 46, 48, 49), while the remaining 11 studies pertain to either a hospital setting (25, 29), multiple settings (23, 24, 31, 33, 37, 44) or do not specify the setting (26, 28, 47).

A total of 12 studies focus on chronic disease management (23, 24, 31, 32, 34, 36–39, 41, 44, 48). While 11 further studies consider preventive care such as screenings (28, 35, 36, 46, 49), immunizations (26, 42) or both (27, 30, 33, 40). The two studies pertaining to hospital care in general (25, 29) are labeled under secondary and tertiary prevention. Two studies focus on activities that correspond to primary, secondary and tertiary levels of prevention (45, 47). From the 27 included studies only one study explicitly addresses quaternary prevention (43).

Both per-diem (29) and episode-based payment (25) are only considered by one study each. Neither of these studies, nor our own semi-structured interviews yielded conclusive support for the claim that these types of reimbursement impacted prevention. Echevin and Fortin (29) observe that adding a per-diem fee in 14 departments at a hospital in Quebec (Canada) increased the average length of stay but had ultimately no impact on the delivery of preventive care. Besides this, none of our interviewees is reimbursed on a per-diem basis, therefore no original empirical evidence was collected on this reimbursement through the interviews of our study. Concerning episode-based reimbursement, Cheng et al. (25) conclude that the effect of DRG (diagnosis-related group) payment in 486 Taiwanese hospitals had no significant impact on healthcare preventable adverse outcomes after discharge. As for empirical findings, our interviewed GPs have experience with episode-based reimbursement in primary care, specifically for chronic disease management. Although interviewed GPs are positive about these programs, the difference in type of episode-based reimbursement (hospital vs. primary care) makes it challenging to draw reliable conclusions on this type of reimbursement.

The remainder of the section focusses on the relation between levels of prevention and the better-documented reimbursement systems: FFS (including multiple payment models FFS vs. salary vs. capitation or different blends of FFS) and P4P, respectively. We wish to stress that the included studies vary greatly in design, which affects the extent to which the results may be interpreted as causal or correlative. Clearly, we do not claim that e.g., “the results of cross-sectional studies are by definition correlative” or that “(quasi-)experiments always facilitate causal conclusions”. The causal nature of the results is not always clear to the reader of these studies, us as reviewers included. To inform our analyses, we make mention of study designs in our synthesis of prior research and our own primary research below.

### FFS, Capitation, and Salary on Primary and Secondary Prevention

Most of the included papers in our review describe FFS-based reimbursement, sometimes in combination with capitation and/or salary-based reimbursement. First, we discuss our findings on FFS vs. salary vs. capitation on primary and secondary prevention.

In a cross-sectional study, Jusot et al. (33) report that FFS is associated with a higher delivery of primary and secondary preventive services (specifically, immunization, and screenings) compared to salary and capitation, suggesting that under FFS, professionals have incentives to increase service volume. These results are coherent with what interview respondents report about FFS incentives. One of the interviewed healthcare purchasing specialists believes that preventive activities that are reimbursed through FFS, such as immunizations, will be stimulated under this reimbursement scheme. Two professionals under FFS acknowledge the incentive to increase production of the reimbursed service as this will lead to increased revenue and cited that when preventive activities, such as patient education, are not reimbursable through a fee, this will act as a disincentive for that type of prevention.

On the other hand, in a longitudinal study and a cross-sectional study respectively, Kiran et al. (36) and Dahrouge et al. (27) find no statistically significant differences between these payment models pertaining to screening (27, 36) and immunizations (27). Both studies suggest that the practice's structure (number of enrolled patients per full-time equivalent GP) and organizational factors (such as working with electronic reminders or team-based care) could be stronger determinants for the delivery of preventive services. Accordingly, lack of time was recurrently mentioned during our interviews (by both salaried professionals and professional under FFS) as a reason for not addressing prevention. Two salaried respondents (one GP and one PT) believe that their provider organization plays a crucial role in stimulating prevention at practice-level by making the necessary resources available for professionals to be able to focus their efforts on prevention, such as extending the length of consultations. Salaried respondents claim they would be open to invest more time in prevention, but the perceived pressure from the provider organization (reimbursed under FFS) to generate revenue is hampering prevention, as one PT illustrates: “*I think it depends on your employer and their vision [.] and whether or not they want to stimulate certain things [.] The fact that my schedule is overloaded is because [employers] have certain ideas on how they want to organize things making them less flexible [.] and this will ultimately compromise quality of care”*. Consistent with this statement, two professionals under FFS demonstrate no desire to increase consultation length (as the reimbursed service pertains to a consultation with a predefined length) nor regard this as an important enough obstacle for prevention that needs to be overcome. GPs reimbursed under a mix of capitation and FFS regard the responsibility placed on them for providing primary prevention as unrealistic. The GP does not think it is feasible to extend consultation length and spend (more) time addressing prevention during consultations, suggesting that in order to stimulate prevention in healthcare, other entities such as the municipal health services should be made responsible for addressing prevention. This way, GPs can focus on curative tasks and not patient education: “*As a GP I would really like to apply my medical knowledge and since obesity is a big social problem, I think I would be seeing people all day long and discussing how we are going to tackle someone's obesity. Well, I don't think I would want to do that, no.”*

On the other hand, an interviewed GP recently changed reimbursement from the mixed capitation and FFS to a population-based capitated payment regarding the first segment of GP care. According to this GP, this shift removed the incentive for (over)production. As the provider no longer profits from providing more consultations, this led this GP to extend the consultations' length: “*Now I know what I earn per quarter; it no longer depends on how often I see my patients. So, I choose to take more time for my patients because I don't have to see thirty patients a day to earn my living […]. What we notice is that we no longer, or less often, have to book double appointments […]. And that we have just enough time to approach [prevention]. At first, I was skeptical about it, because you feel that you are losing money by not being able to claim your consultations. But if I compare my practice's finances with those from practices under the traditional reimbursement, I realize that we are definitely not in a bad position financially.”*

### FFS, Capitation, and Salary on Tertiary Prevention

Concerning disease management, Kiran et al. (36) found that GPs' reimbursement with a greater percentage of FFS presented the lowest improvements in diabetes management while reimbursement mostly composed out of capitated payments achieved the largest improvements in diabetes care. With reference to our own empirical research, one respondent PT believes that FFS hinders prevention by reimbursing professionals for every service provided but with no further incentive to avoid disease development, explore potential risk factors that might be the underlying reason for the patient's health complaint or prevent deterioration of a health condition. Similar to Kiran et al. (36), Liddy et al. (41) observe in a cross-sectional study that practices under FFS showed the greatest gaps in adherence to evidence-based guidelines pertaining to cardiovascular disease care. Capitation and salary were similar to each other in results; While salaried GPs scored significantly higher on glucose level control, capitation was linked to increased weight management and smoking cessation drug prescription when compared to FFS and salary. Pearson's et al. (45) cross-sectional study established that GPs for whom >75% of reimbursement consisted of capitation relative to FFS were three times more likely to provide patient education. In sum, salary, and even more so capitation, rather than FFS, appear to be related to better disease management.

### FFS, Capitation, and Salary on Quaternary Prevention

Our rapid review yielded no results on quaternary prevention under FFS, salary nor capitation. Nevertheless, our interviews suggest that overmedicalization is still prevalent in healthcare. When asked about overmedicalization, respondents under these three reimbursement schemes believe that it is mostly driven by patient demand, not by reimbursement, and claim they run responsible practices as overprovision might have consequences for the patient's health and healthcare expenditure: “*We are always critical about what is necessary, what is medically indicated.”* However, all professionals acknowledge that in some circumstances they might (partially) give in to patients' demands, as a salaried GP illustrates: “*I also try to negotiate a little bit [.] but yeah, I'm not saying I don't do it. Because you also have a future with that patient, in your doctor-patient relationship. [.] I never give [prescription for blood test] without explaining very clearly what you can and what you cannot get with it [.] Because some things just have consequences [.] and then you enter into an unnecessary medicalization process.”*

### P4P on Primary and Secondary Prevention

In the remainder of this section, we present our findings on P4P-based reimbursement in relation to the levels of prevention. First, we discuss primary and secondary prevention.

In an interrupted times series study, Chien et al. (26) examines the effect of a piece-rate P4P bonus for full and timely childhood immunization. Results show that immunization within the P4P program increased at a significantly higher rate than the comparison group. Similarly, Merilind et al. (42) reveals that GPs under P4P achieved the target of 90% coverage rate for all vaccinations while the comparison group only achieved the target rate for one vaccination. Both studies suggest that P4P schemes with a bonus specifically for immunizations can improve immunization rates. Pendrith et al. (46) observed that FFS on its own provided low incentives for the delivery of cancer screening and that a P4P bonus for achieving 60% or 80% screening rates for three types of cancer combined with FFS lead to an increased provision of these screenings. The combination of capitation and the same P4P bonus also presented higher screening rates in comparison to FFS, leading authors to suggest that adding these P4P incentives was associated with higher cancer screening rates. Conversely to these findings, Kiran et al. (35) observed that despite the increase in billing for self-reported provision of cancer screenings leading to larger expenditures there was little or no significant increase in cancer screening rates after the introduction of P4P bonuses for the achievement of different targets in screening rates. Similarly, two other studies observed that cancer screening rates (30) and immunization rates (30, 40) did not suffer significant changes after implementation of a P4P bonus among GPs for the achievement of targets pertaining to the delivery of these preventive services. Both studies hypothesize that these incentives representing 3–4% (30) and <10% of annual income (40) might have been too small to induce the desired changes in practice. Besides this, authors suggest that other aspects such as provider training (30) and lack of provider reminder systems (35) could impact performance. Li et al. (40) question P4P's effect on the quality of (preventive) care and suggest that the introduction of five different indicators simultaneously might decrease the likelihood of physician's response to any of them. Further research on why and how P4P's design features can help increase professionals' response is required (40).

Different studies provide different interpretations of how P4P's components influence professionals' performance. De Walque's et al. (28) results reveal that P4P higher incentives (US$4.59) had a greater and significant impact on indicators such as couples HIV testing, compared to lower incentives (US$0.92) for individual HIV testing. The latter shows little or no significant effect on professionals' performance. In line with De Walque et al. (28), Sicsic and Franc's (49) study observes little impact of a P4P program among French GPs on breast cancer screening rates, concluding that the “low-powered” financial incentive (maximum €245/target) did not have enough leverage to stimulate providers. Contrastingly, Rajkotia et al. (47) propose that practices are not necessarily more responsive to more profitable indicators (such as the survival rate after treatment of HIV-infected children with a $11.20 reward) than to less profitable ones ($4.20), but instead prioritize targets that can be achieved with a lower level of effort, in this case the number of HIV-infected pregnant women initiating antiretroviral therapy ($10 reward) or the number of family planning consultations given to HIV-infected women ($5 reward). Taken together, these studies suggest that both bonus magnitude (28, 30, 35, 40, 49) and required effort (30, 47) are important components in a P4P scheme. Our interviews did not produce results pertaining to the effect of P4P on primary and secondary prevention.

### P4P on Tertiary Prevention

Regarding the effect of P4P on disease management, two studies reveal that Taiwanese diabetes mellitus (DM) patients of P4P-enrolled GPs had higher continuity of care and lower mortality rates (44) and were more likely to receive the P4P-rewarded guideline-recommended DM examinations than patients treated by non-P4P GPs, as presented in a cross-sectional study by Lai and Hou (37). Two longitudinal cohort studies on cardiovascular disease management (23) and diabetes management (32) observe that financially incentivizing disease management check-ups and treatments also resulted in fewer (avoidable) hospitalizations. Chen et al. (23) suggest that P4P success might be due to an easily achievable target concerning the percentage of patients receiving improved quality of care and reaching positive health outcomes for cardiovascular disease. The low baseline rate for improvement (42%) stimulated participation in the P4P program. Hsieh et al. (31) observe that rewarding professionals for process indicators (e.g., control visits and cholesterol and glucose testing) led to no difference in the number of visits nor tests performed. When outcome indicators rewarding improvement in clinical levels (e.g., cholesterol levels) were added to the P4P program, quality of care improved.

Serumaga's et al. (48) interrupted time series study found that rewarding blood pressure control and drug prescription for hypertension disease management did not increase the delivery of these services in a clinically or statistically significant manner, nor were there changes in mortality rates or other hypertension related adverse outcomes. The authors suggest that blood pressure control had already improved before the implementation of P4P and that P4P targets might have been set too low for significant change to take place. Similarly, the results of both Lee et al. (39) and Chen et al. (24) show that the delivery of secondary and tertiary preventive services did not significantly vary between the P4P and non-P4P groups. Chen et al. (24) cited the small bonus size ($3-$30/service/per patient) and low provider participation as probable explanations for low behavioral change in the delivery of three guideline-recommended preventive services and test for Hepatitis B and Hepatitis C patients. Lee et al. (39) stated that future P4P programs rewarding blood pressure and cholesterol level controls should include achievable but at the same time challenging targets to create enough leverage among different practices.

Two of our interviewed GPs believe that performance targets set by the healthcare purchaser limit their professional autonomy and control the way care is delivered without taking other factors into consideration: “*The healthcare purchaser imposes targets on me that I must meet, which may not be feasible at all for a great part of my patients because I have many elderly people or immigrants, for example, and then I have a problem*.” Another GP adds: “*If I prescribe expensive medication once, it is probably because there is medical necessity, which is never accepted (by the purchaser), because then there are again twenty-six conditions that it must meet.”* The interviewees also believe these targets are set primarily in order to reduce healthcare costs and not to increase quality.

In a P4P program financially rewarding providers for conducting all recommended diabetes management actions, LeBlanc et al. (38) conclude that although patients of GPs claiming the bonus received more glucose tests per year and had consequently better GP follow-up, this was ultimately not translated into lower glucose levels for those patients relative to the comparison group. Similarly, Karunaratne et al. (34) prospective longitudinal cohort study examines the management of chronic kidney disease in primary care before and after implementation of P4P bonuses for initial and ongoing management actions such as blood pressure measurement and control. Contrary to Serumaga's et al. (48) findings, in this case blood pressure measurement and prescription medication significantly increased as did costs associated with increased prescribing. However, Karunaratne et al. (34) cannot establish if these events consequently resulted in improved health outcomes, delayed disease progression or decreased mortality. Similarly, in our empirical research, two respondent GPs question the quality improvement incentive P4P programs aims to induce and raised some concerns regarding such incentives of P4P: “*You can also ask yourself whether that [P4P] really improves quality, because you mainly measure whether people have been seen [by the doctor], but whether you will take action or do something with those [blood test] results is the real question.”*

### P4P on Quaternary Prevention

Norman's et al. (43) interview study with GPs is the only study in our research that explicitly investigates P4P's impact on quaternary prevention. GPs under P4P revealed experiencing an incentive to reduce any risk factor and prophylactically treat patients as otherwise it might disturb P4P target achievement. Also, Norman et al. (43) report that GPs acknowledged opting to medicate patients rather than trying non-pharmacological approaches simply to be able to timely achieve P4P targets, and additionally acknowledged that even when indicators went against GPs' inner values, they still complied and strived to achieve targets. These reported incentives are acknowledged by two of our own respondent GPs who disagree with the implementation of P4P and worry that money may become the incentive for action, adding: “*Because then we will do things because you get money for it and not because you actually want to work that way”*.

## Discussion

Our research collected empirical evidence on the relationships between different reimbursement schemes and four levels of prevention. Most of our results relate to P4P, FFS, capitation and salary. We also obtained results on both per-diem and episode-based payment, however neither the rapid review nor our own empirical research could confirm the impact of these types of reimbursements on prevention. Quaternary prevention was addressed by one study only. The integration of findings from both the rapid review and our original empirical research allows us to draw the following main discussion points regarding reimbursement schemes and prevention.

### Salary

The findings of our rapid review on the impact of salary-based reimbursement are ambiguous, associating salary to both higher and lower delivery of preventive services compared to FFS and capitation (33, 41). In previous literature, while Gosden et al. (50) claims salaried professionals want to minimize their personal efforts, Kane et al. (4) proposes that these professionals could be incentivized to engage in preventive care more than professionals reimbursed under FFS. An obstacle experienced by salaried respondents are the incentives from the employer organization and not making the necessary resources available to stimulate prevention, suggesting that the reimbursement of the provider organization or practice might interfere with salaried professionals' behavior toward prevention. Therefore, it should be taken into consideration that these different interactions between the professional and the provider organization can align or misalign incentives which might impact prevention in practice.

### FFS

Concerning FFS, although ambiguous, results suggested that its widely reported incentive to increase production might work in favor of preventive services such as immunizations, eye exams and screening for cancer (33). The corollary is that non-reimbursed activities, longer consultations or less consultations might evoke feelings of loss for these professionals, according to empirical findings. In fact, interviewees acknowledged the fact that when prevention is not as widely reimbursed in fees this poses as an obstacle for the provision of preventive services. More than a decade ago, Ellis and Miller (14) already proposed that activities not reimbursed through fees (such as preventive counseling) can be neglected under FFS. Results also suggest that FFS restricts the delivery of care to predefined standards and does not allow the flexibility to organize care delivery differently, as some forms of prevention might require. Therefore, FFS might work for some forms of prevention, in particular when the prevention activity can be specified as reimbursable under FFS (such as immunizations), but it can be questioned whether it will stimulate professionals to address prevention when the preventive activity requires efforts/services that are not reimbursed through a fee.

### Population-Based Payment

Both PTs and GPs state that, as the patient's medical complaint receives primary attention during consultations and sometimes the consultation is even too short to address the complaint in detail, there is usually not enough time left to address risk factors and address secondary and tertiary prevention. However, opinions of GPs under population-based capitated payment contrast with those of GPs under traditional capitation and FFS reimbursement. A GP under traditional reimbursement considers the responsibility to deliver preventive services by GPs to be unrealistic and therefore does not feel increasing consultation length to be necessary. Instead, the responsibility for prevention should be placed elsewhere. A focus on revenue and profit might be a reason to increase the number of consultations per day and no desire to extend consultation length. On the other hand, GPs under population-based capitation report having altered the delivery of care to facilitate the delivery of prevention through extending consultation length. This is due to a bigger focus on prevention as a cost reduction strategy from capitation incentives and a reduced volume incentive with the elimination of FFS.

Although respondents show that they are aware of the importance of quaternary prevention, they acknowledge that overmedicalization still persists in healthcare due to patients' demands. In case of time constraints, professionals might be likely to resort to unnecessary prescriptions or referrals in detriment of providing important information, comprehensively discussing alternative approaches that mitigate overmedicalization, compromising quaternary prevention.

### P4P

Both our rapid review and our interviews' ambiguous findings question P4P's promise to improve healthcare quality through better prevention. Studies show that the achievement of targets for performing disease management examinations is not necessarily translated into better patient outcomes or effective secondary and tertiary prevention (34, 38). Similarly, Flodgren et al. (51) already concluded in their systematic review that P4P effectively managed to change professional's practice, however no effect on patient outcomes is subsequently observed. Empirical results also report how P4P's metrics might steer professional's behavior and how this might conflict with what is best for the patient. The risk of losing a bonus might trigger professionals to circumvent factors standing in the way of achieving targets. The results of our rapid review underscore the importance of different P4P design features in stimulating behavior. However, the role of bonus size and level of effort in responsiveness to an indicator are discordantly described. While Gavagan et al. (30), Li et al. (40), De Walque et al. (28), Chen et al. (24), and Sicsic and Franc (49) cite the importance of bonus size, Rajkotia et al. (47) claim that the level of effort necessary to reach a target is the most important determinant of behavior. Serumaga et al. (48) and Chen et al. (23) claim that targets should be low enough to motivate professionals while Chen et al. (24) and Lee et al. (39) report that, in order to make P4P cost-effective, targets should not be set too low and easy to achieve. On the other hand, Hsieh et al. (31) claim that only when incentives for outcome indicators are added to the P4P program, improvements in quality of care are observed. Taken together, these studies suggest that bonus magnitude, required effort and type of indicator are important components in a P4P scheme. Even though our research did not further investigate the role of these components, our results suggest this should be considered as it most likely will influence a reimbursement's effectiveness toward prevention.

Regarding quaternary prevention, Norman et al. (43) show how P4P can lead professionals to resort to (over)medicalization to achieve targets, compromising this level of prevention. Karunaratne et al. (34) show that P4P leads to a rise in medication prescription and costs related to increased prescription with no further improvement in health outcomes. In line with these results, interviewees are critical about P4P and worry that money may become the incentive for action, and this may “crowd out” intrinsic motivation to deliver efficiency and quality in healthcare. While P4P is promoted by purchasers, it is considered disruptive by professionals, who suggest it might trigger different unintended behaviors in professionals that ultimately hinder effective prevention at different levels.

### Per-Diem and Episode-Based Payment

Both per-diem and episode-based payment are only considered by one study each and neither can claim these reimbursement schemes impact prevention. None of our interviewees has experience with per-diem reimbursement. Interviewees stated that primary care episode-based payment creates incentives to better organize disease management and actively monitor patients so as to avoid complications from which the provider could incur additional costs. This type of episode-based payment is a form of prospective reimbursement, with revenues known upfront and hence there is a strong incentive for cost avoidance.

## Conclusions

The benefits of preventive health services are widely recognized. However, delivery of prevention services encounters numerous obstacles in healthcare. It is argued that reimbursement schemes play an important role in both hindering and stimulating the provision of healthcare services (13). Nevertheless, there has been little focus on how reimbursement schemes could specifically contribute to the delivery of preventive health services.

Our research provides insights into how different types of reimbursement (e.g., fee-for-service, or pay-for-performance) impact healthcare professionals' behavior; stimulating or hindering their efforts to address prevention. We distinguish between four levels of prevention, ranging from avoiding disease onset, allowing early diagnose and reducing disease impact to protecting patients from receiving redundant, unnecessary care. We find that not one ideal reimbursement scheme exists, providing incentives that stimulate (or hinder) prevention at all its levels. There are, however, certain types of reimbursements that work well for certain types of preventive care services. For example, the volume incentive from FFS could be beneficial for some levels of prevention when clearly specified preventive actions are concerned (such as immunization, as an example of primary prevention or screenings as an example of secondary prevention). On the other hand, population based capitated reimbursement might facilitate the delivery of some forms of prevention that are more difficult to specify as a reimbursable service, or for which lack of time poses as an obstacle under other reimbursement schemes, allowing the flexibility to alter the delivery of care. We also discuss P4P, as this is prominent in both our literature review as well as amongst our interviewees. As our study empirically reported, P4P's incentives might have unintended consequences for professionals' intrinsic motivation. P4P's criteria for medication prescription is an example of how what is being measured and therefore reimbursed for could influence a professional's practice away from what they initially intended. Additionally, the achievement of P4P's targets does not always imply better health outcomes (34, 38). Besides this, our study also describes how the pressure to (timely) achieve targets arising from this type of reimbursement can lead professionals to resort to (over)medicalization and discard other approaches that could better fit the patient's needs, compromising quaternary prevention (43).

### Strengths and Limitations

The strength of our study lies in the incorporation of evidence from both a rapid review of the literature and interviews with professionals to help consolidate results and achieve a more comprehensive explanation of how reimbursement schemes can affect prevention. Furthermore, this research raises awareness on overmedicalization by contemplating quaternary prevention as the fourth level of prevention, contributing with a more overarching definition of prevention. Although efforts were made to mitigate bias, this research has a few limitations which will be outlined in this section. These limitations could provide additional guidance for future research.

First regarding the rapid review. Despite efforts to test and strengthen our search strategies, it is entirely possible that studies to prevention have unintentionally been omitted, due to them not being described with the term “prevention.” Quaternary prevention is still a relatively new concept and therefore strategies to reduce medical overuse might not be perceived and labeled as (a level of) prevention. In addition, despite the advantages of rapid review methodology, drawbacks must be acknowledged, i.e., the process of data collection, selection and analysis was primarily performed by one reviewer. This may have compromised the study selection procedure and consequently the reliability of results. Also, unlike more traditional systematic literature reviews, rapid reviews rely on narrative analysis and synthesis rather than meta-analysis of the included studies (18). Although we argue that our rapid review fits our research aim well, a more traditional systematic approach would be better suited for more detailed and quantitatively sophisticated meta-analysis of the effectiveness of payment models in relation to e.g., case mix of patients. Meta-analysis would also allow for further assessment of the quality of the results and the level of evidence provided by the included studies.

As addressed by some studies included in our review, the effectiveness of any reimbursement scheme is likely to be affected by the generosity of the payment and not solely the type of reimbursement. However, not all studies from our rapid review disclose the reimbursed amounts in analogous ways, making it impossible to compare payment generosity and draw conclusions on this matter. Therefore, we did not investigate this matter in our review which could have provided additional and valuable insights to our research. We recognize this as a limitation of our study.

We acknowledge that there may be various other factors that interact with reimbursement scheme to impact delivery of prevention services. These can be factors related to the healthcare delivery model of a country, the level of investment in healthcare in a country, social, economic or cultural differences between countries, or differences between countries or regions in support structures offered to healthcare professionals other than reimbursement. We summarize the evidence at a relatively high level of abstraction, between reimbursement scheme and delivery of prevention services only, and cannot account for all differences between countries. Moreover, our rapid review is skewed toward studies executed in OECD countries, more specifically North America, and countries with a National Health Insurance (NHI) model. Jusot et al. (33) have shown, in their 14-country study, that factors related to reimbursement were most strongly related to utilization of preventive services, with other system-level factors, like capacity or structure, playing a lesser role. This lends credibility to our assumption that reimbursement scheme is a very important factor, even if it may interact with other factors. Still, looking at reimbursement separately is a limitation, and we recommend future research to also study interactions with other factors.

Concerning the empirical research, interviews were planned to be conducted face-to-face, however, due to local restrictions regarding the COVID-19 outbreak at the time of this study's data collection, interviews had to be conducted via telephone. Furthermore, some of the initially targeted respondents had to cancel their participation. The initial intention was to conceptualize a “theoretical sample” by means of literature review and subsequently select respondents based on that theoretical sample. Both research setting and respondents had to be rearranged in a short period of time and respondents had to be recruited through “convenience sampling” and “snowball sampling” (52), limiting the opportunities to draw a varied sample of respondents as initially intended. Medical specialists and patients, who could have added valuable insights, were not interviewed. Due to the qualitative research design and convenience sampling of a limited number of respondents, this research could lack representativeness and external validity.

Neither the rapid review nor the empirical research provided ample insights on all relevant reimbursement schemes. On the one hand because our sample did not include respondents who experienced all types of reimbursement, and secondly because the rapid review identified relatively many studies on certain reimbursement schemes but less so on others. Future research should target also other respondent samples for a more comprehensive understanding of how reimbursement may affect prevention. Besides this, most respondents were paid under a mix of reimbursements which makes it difficult to assess their isolated effect and can compromise results.

The fact that healthcare professionals' behaviors might be stimulated or hindered by incentives from different types of reimbursement schemes could be regarded as in conflict with the oath of ethics concerning non-maleficence. During the interviews it was noticeable that some respondents were more hesitant to talk about possible incentives altering their behavior in terms of under- and/or overprovision of care and might have held back valuable information.

Finally, our rapid review identified only one study on quaternary prevention and more research is needed on this level of prevention and how different reimbursement schemes impact quaternary prevention.

## Author Contributions

EZ conceived the idea, performed the review and interviews, and wrote the first draft of the manuscript. ER and HE assisted the rapid review and contributed to the data interpretation process. All authors wrote and edited the manuscript and approved the submitted version.

## Conflict of Interest

The authors declare that the research was conducted in the absence of any commercial or financial relationships that could be construed as a potential conflict of interest.

## Publisher's Note

All claims expressed in this article are solely those of the authors and do not necessarily represent those of their affiliated organizations, or those of the publisher, the editors and the reviewers. Any product that may be evaluated in this article, or claim that may be made by its manufacturer, is not guaranteed or endorsed by the publisher.

## Appendix

### Topic List for Interviews

**Introduction**

- Thank respondent for their availability
- Briefly describe the purpose and logistics of this interview
- Ask for permission to record interview for the purpose of transcription
- Start the interview by first collecting the respondent's professional information.

**Views on prevention**

- Professional's definition of prevention
- Prevention in (daily) practice
- Roles in the provision of prevention
- Prevention and the patient

**Reimbursement and prevention**

- Reimbursement of preventive services
- Preventive care programs
- Encouragements/obstacles for the delivery of preventive services
- Healthcare purchaser's role

**Quaternary prevention/Medicalization**

- Benefits/risks of prevention for the patient
- Overmedicalization in healthcare
- Causes for overmedicalization
- Healthcare professionals and overmedicalization
- Strategies to mitigate overmedicalization

**Conclusion**

- Guarantee respondent's anonymity
- Ask permission to use quotes
- Thank the respondent.
