# Supplementary material for: The Linkages Between Reimbursement and Prevention: A Mixed-Methods Approach
Source: Front Public Health. 2021 Oct 27;9:750122. doi: 10.3389/fpubh.2021.750122 (PMC8578935; doi:10.3389/fpubh.2021.750122)
Supplement: Supplementary file 1 [file Data_Sheet_1.docx]

Supplementary Material

# Supplementary Data 1 - Rapid Review Search String

The search was conducted using the following search string:

*(provider* OR professional* OR physician* OR clinician* OR practitioner* OR doctor* OR specialist*) AND (incentive* OR behavior* OR behaviour* OR prevention) AND (payment* OR reimbursement* OR remuneration*)*

**Supplementary Data 2 – Table with additional information of included studies in rapid review**

|  |  |  |  | |
| --- | --- | --- | --- | --- |
| **Author(s), Year** | **Reimbursement** | **Indicators** | **Results and conclusions** | |
| **Chen et al., 2011** | P4P Bonus (3,5% above reimbursement: maximum $16,000/year) Performance based on physician level | High-quality care (i.e., receipt of at least 1 laboratory test for LDL and at least 1 prescription for a statin within a 1-year period) among patients 18 to 75 years of age with cardiovascular disease. | Treatment by a P4P participating provider was associated with a greater increase in the receipt of high-quality care over time. The effect of the P4P program steadily grew and reached 15% difference between the two groups after 6years. | |
| **Chen et al., 2016** | P4P Bonus for completion of visits $3 /visit/patient + additional bonus for further screening, referral, early detection of abnormalities per patient ($15 - $30) Performance based on physician level | The utilization of three guideline-recommended preventive services test for Hepatitis B and Hepatitis C patients (twice annual visits; Abdominal examinations; laboratory tests) | Following the implementation of the P4P program, the magnitude of change in recommendation of preventive services was present but was relatively small. Financial incentives might play a role in physicians' adherence to guidelines and therefore improve quality of care, however, due to the small improvement more stronger efforts will be required in the future. | |
| **Cheng et al., 2012** | Episode-based (DRG) vs FFS | Changes in medical service content: length of stay (LOS) and intensity of care (the number of orders for medications, the number of orders for diagnosis or treatment, and the number of orders for special materials or devices) and healthcare outcomes (likelihood of emergency department (ED) visits within three days after discharge, the likelihood of readmission within 30 days after discharge, and the likelihood of mortality within 30 days after discharge) | Findings revealed that the introduction of the DRG scheme resulted in shortened LOS and decreased intensity of care. No significant changes were found concerning health care outcomes. | |
| **Chien et al., 2010** | P4P Piece-rate bonus (15-25% above reimbursement) Bonus for immunizations $100/patient and extra bonus $100/patient for timeliness Performance based on practice level | Full and timely immunization of 2-year-olds | Immunization rates rose at a significantly, yet modestly, higher rate than the comparison group. Authors suggest that the P4P program with the appropriate administrative supports could be effective at improving childhood immunization rates | |
| **Dahrouge et al., 2011** | FFS vs Salary vs New capitation (Capitation + 10% FFS) vs Traditional/established capitation | Six preventive maneuvers: Influenza immunization; Cervical cancer screening; Breast cancer screening; Colorectal cancer screening; Visual impairment screening; Auditory impairment screening | Prevention scores were lower in practices in the fee-for-service and established capitation models compared to the new capitation model. However, these results were not statistically significant after inclusion on organizational factors in the analysis. So, practice structure rather than funding arrangements was the primary determinant of the delivery of evidence-based preventive health care. | |
| **De Walque et al., 2015** | P4P Bonus (different amounts for different services: individual testing US$0.92; couple testing US$4.59) Performance based on practice level | Two P4P indicators: Individual HIV testing and counseling AND couple HIV testing and counseling | There was no effect on single individuals. There was a positive and statistically significant impact for individuals in couples, consistent with the fact that the P4P scheme strongly encouraged couple and partner as the bonus for testing couples was higher than the bonus for testing individuals. Concluding that, in general, services for which prices were higher and providers found easier to implement had the larger responses. | |
| **Échevin and Fortin, 2014** | Per-diem | Risk of re-hospitalization | As results show that LOS increased by 0.28 days while risk of re-hospitalization did not seem to be affected, authors suggest that as risk of re-hospitalization did not improve it is not likely that professionals spend more time treating patients and therefore improving their health. The hypothesize that professionals on the new per-diem reimbursement may spend more time on non-clinical activities which would be likely neglected under a traditional FFS and recommend further research to be conducted on the matter. | |
| **Gavagan et al., 2010** | P4P Bonus (max $12,000 annually/physician ($4000/target = 3-4% annual income)) Performance based on physician level | Targets in cervical cancer screening, mammography, and pediatric immunizations. | Overall, there was no clinically significant effect of P4P incentives increase the delivery of preventive services. Many other factors are related to the performance of prevention in primary care offices, including availability of information technology, provider factors and training, and reimbursement. Future research should gather better evidence. | |
| **Hsieh et al., 2016** | P4P Diabetes program. Phase 1 process indicators - Bonus for process indicators ($30.00 - $75.00/visit); Phase 2 - Bonus ($30.00/visit) for process indicators conditional on performance of outcome indicators. Ranking (top 25% performing physician get extra bonus)  Performance based on physician level | Process measures (provision of different laboratory tests) and Outcome measures (changes in those values between baseline and follow-up). | Process measures did not significantly improve between both phases as they were rewarded in both phases. When additional outcomes measures were introduced in the second phase, more improvement was found in patients’ health. Non-incentivized measures did not show negative unintended consequences. However, authors point that caution is required regarding the possible consequence of "teaching to the test". | |
| **Iezzi et al., 2014** | P4P "low powered incentives" Performance based on physician level | The number of avoidable hospitalizations per year per GP. Given that timely, quality treatment should help prevent such hospitalizations, this adverse event is an indicator of poor quality of care. | Results imply that diabetic patients followed by GPs receiving a higher share of their pay through P4P are less likely to experience avoidable hospitalizations for diabetes-related diseases. Findings indicate that financial transfers may help improve the quality of care, even when they are not based on the ex-post verification of performance. So, there are cases where low-powered incentives may improve patient's condition even though ex-post monitoring of performance is not strictly enforced. | |
| **Jusot et al., 2011** | FFS vs Capitation vs Salary | Four preventive services: Flu vaccination; Eye examination; Mammography; Colonoscopy | Payment schemes for providers appear to have a significant influence on the provision and utilization of preventive and health services. In systems where doctors are paid by FFS, the utilization of all health services are higher. This is coherent with economic theory that under fee-for-service doctors have incentives to increase the volume of their services. Capitation is often used as an alternative to provide focus on primary care without dropping cost control, but there seems to be a risk in these schemes of under-referring for secondary prevention (such as eye exams and colonoscopy). | |
| **Karunaratne et al., 2013** | P4P – Quality and Outcomes Framework (QOF) (representing 25% of income) Performance based on practice level | The mean systolic and diastolic blood pressures together with the prescription of antihypertensive medication analyzed over three time periods | Results show that P4P was associated with a significant blood pressure reduction together with an increase in prescription of several blood pressure agents. Authors conclude that the P4P program lead to a significant improvement in blood pressure and as the cost associated in term of increased prescribing were modest, authors claim that the P4P program could be cost-effective if the potential health gains and improved outcomes in the future could offset these costs. | |
| **Kiran et al., 2014** | P4P Bonus (max $8.400/annually - 3% of gross income) for reaching screening targets Performance based on physician level | Colorectal-, cervical- and breast cancer screening rates | The implementation of a P4P scheme was associated with little or no increase in cancer screening rates despite relatively large expenditure and very good uptake of incentives among primary care physicians. There was no significant change in breast and cervical cancer screening rates. Authors recommend policy makers to consider other strategies to improve cancer screening rates. | |
| **Kiran et al., 2015** | Different combination of FFS and capitation - 3 forms of payments in PCMH: Enhanced FFS (15% capitation; 80% FFS and 5% incentives) Non-team-based capitation (70% capitation; 20% FFS and 10% incentives) and Team-based capitation (70% capitation; 20% FFS and 10% incentives + eligible for funding to hire allied health professionals) | The delivery of evidence-based testing for diabetes and the delivery of recommended screening for cervical, breast and colorectal cancer | Results suggest that the shift to capitation may contribute to (moderate) improvements in diabetes care. The effects on cancer screening are less clear. Improvements in diabetes care were most marked when capitation payment to physicians was coupled with interprofessional care teams. Authors conclude that physician payment reform and team-based care have the potential to improve chronic disease management and prevention although they were not able to infer causation. | |
| **Lai and Hou, 2013** | P4P Diabetes Program. Physicians receive fees for enrolling patients in program. Incentives for process indicators and Outcome indicators  Performance based on physician level | Guideline adherence to: hemoglobin A1c, blood glucose, lipid, serum creatinine, alanine transaminase, urinalysis, and eye examinations. | Patients enrolled in the DM-P4P program were more likely to receive all guideline-recommended tests/examinations than patients treated by non-P4P physicians. Patients who were not enrolled in the program but who were treated by DM-P4P-participating physicians were more likely to receive three of the seven recommended tests/examinations than were those treated by non-P4P physicians. Authors suggest that participating physicians might delivered better outcomes regardless of the patient being enrolled. In conclusion, physicians participating in the DM-P4P program provided significantly more guideline recommended tests and examinations to their patients. | |
| **LeBlanc et al., 2016** | P4P Bonus (annually CAN$83.83/patient for completing all indicators) Performance based on physician level | Patients receiving (at least) two A1C (glycated hemoglobin) tests + achieving glycemic control (lower clinical values) | Despite being associated with greater odds of receiving at least recommended amount of two A1C tests, the P4P program was not related to an improvement in glycemic levels of diabetes patients. Authors argue that the results should be reassessed in the future to verify if the glycemic clinical values diminished, as A1C levels might take longer to improve. | |
| **Lee et al., 2011** | P4P - QOF (representing 25% of income) Performance based on practice level | Mean systolic and diastolic blood pressure and cholesterol levels | Results show that there was an initial step change improvement in quality of care after the implementation of P4P, however this effect is mainly due to practices that had a worse baseline performance and that it had little or no effect on practices already achieving the targets before the implementation of QOF. Authors suggest that when targets are too easy to achieve/when practices are already achieving the target before implementation of QOF this might produce little gain in quality for the money spent and will largely reward those already operating at a high level. These factors should be taken in consideration when implementing such P4P programs. | |
| **Li et al., 2014** | P4P Bonus for reaching all 5 targets = $11.000 + bonus for scheduling appointments for eligle patients = $11.000 - TOTAL (maximum ($22.000) <10% annual revenue) Performance based on physician level | Five preventive primary care services: childhood immunizations, adult immunizations, cervical cancer screening, breast cancer screening, colorectal cancer screening | There was a modest improvement in performance for cervical cancer screening, breast cancer screening, cervical cancer screening and adult immunizations but no response/improvement regarding childhood immunizations. This mixed and modest GP response led authors to conclude that caution should be taken when claiming P4P are effective in stimulating the delivery of preventive services and plea for future research to contemplate two understudied factors: why do GPs respond (or not) to incentives and the impact of alternative P4P designs (which features induce large responses). | |
| **Liddy et al., 2011** | FFS (mainly FFS) vs Blended Capitation (mainly capitation) vs Salary | Score of each practice’s adherence to ten evidence-based guidelines for cardiovascular disease care (dyslipidemia, diabetes, chronic kidney disease, hypertension, weight management, and smoking cessation care). | Results present important differences in the quality of cardiovascular care delivery among the three primary care reimbursement models. Blended capitation practices provided superior care in the areas of smoking cessation care and waist circumference management, while diabetes monitoring was highest in salaried practices. FFS practices had the greatest gaps in care, especially concerning diabetes care and waist circumference management. However, authors propose that these results are most likely due to a combination of various factors such as individualized characteristics of every practice which influence care delivery. Nevertheless, authors suggest reforms to move away from the traditional FFS practice. | |
| **Merilind et al., 2015** | P4P  (2-4% of GPs reimbursement) Performance based on physician level | Childhood immunization rates | Although there was an improvement in both groups during the observation period, GPs joined to the P4P program had better immunization coverage rates than GPs not joined. GPs not joined to the P4P program were below the immunization coverage rate in all vaccinations. Concluding that the P4P program is attractive for GPs, encouraging them to devote extra efforts to receiving a bonus. | |
| **Norman et al., 2014** | P4P - QOF Bonus for quality targets pertaining clinical care, practice organization and patient experience(representing 25% of income). Performance based on practice level | Professionals’ opinions | This study suggests that QOF has affected GPs’ medical practice in the UK.  Results suggest QOF has the potential to (over)medicalize due to increased tendency to prescribe medications rather than non-pharmacological interventions as life-style changes do not always reach its results within QOF timescales. GPs’ behavior is diverted toward reaching targets and generating income, as professionals admit that despite regarding some targets as “inappropriate”, they still strive to achieve them. | |
| **Pan et al., 2017** | P4P - Diabetes Program Performance is based on 4 indicators - final achievement grade places physician in ranking. Top 25% receive additional bonus. Performance based on physician level | Four indicators: Rate of patients who completed regular follow-ups (at least 3 visits/year); Rate of patients whose glycated hemoglobin (A1C) was lower than 7%; Rate of patients whose glycated hemoglobin (A1C) was higher than 9,5%; Rate of patients whose low-density lipoprotein (LPD) was higher than 130 mg/dL. The achievement rate of each indicator was then summed and became the final achievement grade. | Results show that the score of the P4P participants was 0.227 higher than that of the nonparticipants. P4P participants had a lower HR (hazard ratio) of mortality and higher physician continuity. Authors therefore conclude that the P4P program is an important policy as it could increase survival and/or improve treatment outcomes in specific diseases, specially chronic diseases such as diabetes. Health policy makers should evaluate the possibility of implementing P4P programs to treat other chronic diseases. | |
| **Pearson et al., 2013** | Population-based capitated payments at practice level (different % of capitation). Different levels of capitation. The proportion of revenue received through capitation: 4 levels of capitation: <25%, 26% to 50%, 51% to 75%, and >75% | Patient education provided or ordered at the time of the medical visit. | Results show that practices that received >75% of their revenue from capitation produced a larger proportion of visits that involved patient education compared to practices with lower levels of capitation. Practices receiving >75% revenue through capitation were more than 3 times as likely to involve patient education during visits. Authors conclude that capitated payments are associated with increased delivery of patient education. | |
| **Pendrith et al., 2016** | **P4P** effect: FFS vs FFS+**P4P** vs Capitation+**P4P** Bonus ($220 (60%) - $2,200 (80%)) Performance based on practice level on physician level | Cervical cancer screening rates | Results show that FHG (FFS + P4P) presented the highest screening rates, followed by FHO (capitation + P4P) and FFS. Although FHG (FFS + P4P) had statistically significant higher screening rates than FHO (capitation + P4P) the difference was modest. GPs practicing in FHG (FFS + P4P) and FHO (capitation + P4P) had significantly higher screening rates compared to FFS alone. The difference in screening rates between FFS vs FHG and FHO suggest the addition of P4P is associated with higher screening rates. | |
| **Rajkotia et al., 2017** | P4P Bonus ($0.10-$11.20 per target per patient) Performance based on practice level | 18 indicators on the provision of HIV, mother to child HIV transmission (PMTCT) and maternal/child health (MCH) services. | A positive effect was observed in P4P practices pertaining HIV and MCH services compared to control practices. The majority of the 18 indicators were responsive to P4P. Most adult HIV indicators did not respond to the P4P incentives or had low responsiveness. There was no statistically significant relationship between high bonus size and responsiveness to indicator. There was a statistically significant relationship between lower level of effort and higher responsiveness to indicator. Therefore, authors conclude that P4P is an effective strategy to be applied in healthcare, however it is essential to select indicators that will respond best within the context. | |
| **Serumaga et al., 2011** | P4P - QOF (representing 25% of income) Performance based on practice level | Targets on the delivery high quality of care for hypertension patients: blood pressure over time, rates of blood pressure monitoring, blood pressure control, and treatment intensity at monthly intervals for baseline and 36 months after the implementation of P4P. | No changes in blood pressure monitoring level were observed. P4P had no effect on the cumulative incidence of stroke, myocardial infarction, renal failure, heart failure, or all-cause mortality in both subgroups. Considering the results and the fact that good quality of care for hypertension was stable or already improving before the introduction of the P4P program authors conclude that the P4P program had no discernible effects on processes of care or on hypertension related clinical outcomes. Financial incentives may not be enough to improve quality of care and outcomes for hypertension (and other chronic conditions). | |
| **Sicsic and Franc, 2016** | P4P Bonus - maximum €245/target (80% screened) Performance based on physician level | Breast cancer screening rate | The P4P program had no significant impact on breast cancer screening. Possibly due to multiple obstacles faced by GPs (socioeconomic inequalities, lack of information concerning the achievement of screening, current debates about the relevance of mass screening). Therefore, the P4P program and respective low-powered incentives may not provide enough leverage to increase the uptake of screening | |
|  |  |  |  |  |

**Supplementary Data 3 – Table of Empirical Research’s Respondents Characteristics**

|  |  |
| --- | --- |
| **Respondent Code** | Respondent’s Characteristics **+ Reimbursement** |
| **GP1** | General Practitioner  **Salary**  Works at practice reimbursed through capitation + fee-for-service + EBP + P4P agreements |
| **GP2** | General Practitioner  Practice Owner  Owns practice reimbursed through **population-based payment + EBP + P4P agreements** |
| **GP3** | General Practitioner  **Salary**  Works at practice reimbursed through capitation + fee-for-service + EBP + P4P agreements |
| **GP4** | General Practitioner  Practice Owner  Owns practice reimbursed through **capitation + fee-for-service + EBP + P4P agreements** |
| **PT1** | Physical Therapist  **Salary**  Works at practice reimbursed through fee-for-service |
| **PT2** | Physical Therapist  Practice Owner  Owns practice reimbursed through **fee-for-service + P4P agreements** |
| **PT3** | Physical Therapist  **Salary**  Works at practice reimbursed through fee-for-service + P4P agreements |
| **PT4** | Physical Therapist  **Fee-for-service**  Works at practice reimbursed through fee-for-service |
| **PE1** | Project Manager Prevention at Health Insurance Company  Purchaser /Prevention Expert |
| **PE2** | Senior Intelligence Analyst at Health Insurance Company  Purchaser /Prevention Expert |
|  |  |

# Supplementary Data 4 - Code groups from semi-structured in-depth interviews’ analysis


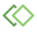
 Capitation **(3 codes)**


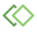
 Efforts made by professionals towards prevention **(9 codes)**


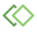
 Episode-based – Ketenzorg **(5 codes)**


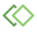
 Fee for service **(26 codes)**


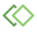
 GP traditional reimbursement **(19 codes)**


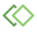
 Need for more attention for prevention / Still a lot to achieve in term of prevention **(1 code)**


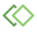
 Obstacles for prevention **(31 codes)**


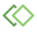
 Pay for Performance **(24 codes)**


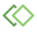
 Population-based payment **(35 codes)**


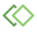
 Professionals perception about own role in prevention **(2 codes)**


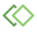
 Reasons for overmedicalization **(11 codes)**


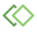
 Reimbursement schemes and prevention **(9 codes)**


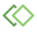
 Relation purchaser – provider **(11 codes)**


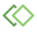
 Salary **(24 codes)**


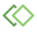
 Shared savings **(20 codes)**


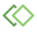
 Strategies to stimulate prevention **(18 codes)**


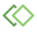
 Views on overmedicalization **(9 codes)**


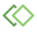
 Views on the importance of prevention **(5 codes)**


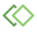
 Ways to mitigate overmedicalization **(14 codes)**
